# Surface Subsidence Analysis by Multi-Temporal InSAR and GRACE: A Case Study in Beijing

**DOI:** 10.3390/s16091495

**Published:** 2016-09-14

**Authors:** Jiming Guo, Lv Zhou, Chaolong Yao, Jiyuan Hu

**Affiliations:** 1 School of Geodesy and Geomatics, Wuhan University, Wuhan 430079, China; jmguo@sgg.whu.edu.cn (J.G.); clyao@whu.edu.cn (C.Y.); plgk@whu.edu.cn (J.H.); 2 Key Laboratory of Precise Engineering and Industry Surveying of National Administration of Surveying, Mapping and Geoinformation, Wuhan University, Wuhan 430079, China; 3 Guangxi Key Laboratory of Spatial Information and Geomatics, Guilin University of Technology, Guilin 541004, China

**Keywords:** multi-temporal InSAR, GRACE, surface subsidence, Beijing, groundwater

## Abstract

The aim of this study was to investigate the relationship between surface subsidence and groundwater changes. To investigate this relationship, we first analyzed surface subsidence. This paper presents the results of a case study of surface subsidence in Beijing from 1 August 2007 to 29 September 2010. The Multi-temporal Interferometric Synthetic Aperture Radar (multi-temporal InSAR) technique, which can simultaneously detect point-like stable reflectors (PSs) and distributed scatterers (DSs), was used to retrieve the subsidence magnitude and distribution in Beijing using 18 ENVISAT ASAR images. The multi-temporal InSAR-derived subsidence was verified by leveling at an accuracy better than 5 mm/year. Based on the verified multi-temporal InSAR results, a prominent uneven subsidence was identified in Beijing. Specifically, most of the subsidence velocities in the downtown area were within 10 mm/year, and the largest subsidence was detected in Tongzhou, with velocities exceeding 140 mm/year. Furthermore, Gravity Recovery and Climate Experiment (GRACE) data were used to derive the groundwater change series and trend. By comparison with the multi-temporal InSAR-derived subsidence results, the long-term decreasing trend between groundwater changes and surface subsidence showed a relatively high consistency, and a significant impact of groundwater changes on the surface subsidence was identified. Additionally, the spatial distribution of the subsidence funnel was partially consistent with that of groundwater depression, i.e., the former possessed a wider range than the latter. Finally, the relationship between surface subsidence and groundwater changes was determined.

## 1. Introduction

Surface subsidence is the main regional environmental geological disaster in the plain areas of China that may negatively affect the safety of infrastructure and human life [1]. In China, more than 70,000 km^2^ in seventeen provinces have been affected to varying degrees by surface subsidence [2,3]. Beijing, as an international metropolis located in the northwest edge of the North China Plain, is one of the most serious areas in China suffering from surface subsidence [4]. Surface subsidence in Beijing was first reported in the 1950s, and it has developed rapidly during recent decades. By the end of 2010, the area of surface subsidence, with a cumulative subsidence of more than 50 mm in Beijing, exceeded 4200 km^2^, and the maximum cumulative subsidence in the significant subsidence area reached 1233 mm [5]. Meanwhile, the annual average subsidence rates have exceeded 100 mm/year in some areas with serious subsidence, such as in Chaoyang and Tongzhou Districts. More than two-thirds of the water demand in Beijing is supplied by groundwater, and the declining groundwater level due to its over-exploitation is the main cause of surface subsidence formation and development in Beijing [6,7]. Recently, rapid economic development, population growth and the sharp increase in mining groundwater have made the surface subsidence in Beijing increasingly serious. Therefore, monitoring and analyzing the spatial distribution and the state of surface subsidence activity in Beijing are crucially important for disaster prevention and the sustainable development of urbanization.

For monitoring and analyzing surface subsidence, conventional monitoring methods (e.g., leveling, Global Navigation Satellite System (GNSS), and layer-wise mark measurements [8]) can retrieve the deformation of a monitoring point with high temporal resolution and measurement accuracy, although the high cost and low spatial resolution of these methods make it difficult to efficiently monitor and analyze regional subsidence.

Recently, the interferometric synthetic aperture radar (InSAR) technique has been recognized as an effective low-cost method for monitoring and analyzing surface subsidence and has been successfully used in many regions, such as Las Vegas, Nevada [9], Iran [10] and western Indonesia [11]. InSAR can overcome the drawbacks of conventional methods (i.e., high cost and low spatial resolution) and can retrieve surface deformation information over large areas and with high spatial resolution. Conventional differential interferometric synthetic aperture radar (DInSAR) can detect regional-scale surface subsidence with a centimeter-to-millimeter accuracy [12]. Due to its all-weather capability of obtaining deformation information, this technique has been used widely, such as in measuring earthquake deformation [13,14], detecting and monitoring landslides [15,16], and mapping and quantifying urban surface subsidence [17]. InSAR techniques have been recognized as effective low-cost methods for anticipating and monitoring natural hazards [18]. However, DInSAR is susceptible to atmospheric delay and temporal and spatial decorrelation, reducing the competency of the technique for long-term interval surface monitoring with high precision.

To overcome the shortcomings of DInSAR, the Persistent Scatterer Interferometric Synthetic Aperture Radar (PS InSAR) technique [19] has been proposed. This technique can overcome decorrelation and atmospheric delay problems by identifying and analyzing point-like stable reflectors (PSs), such as buildings, rock outcrops and railway-related objects. PS InSAR can monitor terrain deformation with millimetric accuracy [19,20], although it requires many SAR images (generally more than 25 scenes). Additionally, another approach, referred to as the Small Baseline Subset Interferometric Synthetic Aperture Radar (SBAS InSAR) technique [21], extracts deformation information via analyzing distributed scatterers (DSs) with high coherence. SBAS InSAR can effectively mitigate the decorrelation phenomena based on an appropriate combination of interferograms produced by data pairs characterized by short temporal and spatial baselines [21]. Compared with PS InSAR, SBAS InSAR can retrieve deformation information with fewer SAR images, and nonlinear deformation information is more clearly captured. Although SBAS InSAR works well in monitoring deformations occurring at a relatively large spatial scale (pixel dimensions in the order of 100 m × 100 m are typical), it is not appropriate to analyze local deformations, such as single buildings or structures [22]. PS InSAR is good at detecting PSs, whereas SBAS InSAR works well for detecting DSs. Some other methods, aiming to monitor deformation in different conditions, have been presented. For example, the Intermittent Small Baseline Subset (ISBAS) technique [23,24], which provides greater spatial coverage and thereby increases its usefulness for rural areas, has been used successfully for monitoring the coalfields in the UK; an improved SBAS algorithm [25], which has extended the SBAS implemented in the StaMPS software, has been able to assess the deformation in mountainous regions; the Quasi-PS (QPS) technique [26], which was proposed for detecting targets with partial coherence, has been applied successfully in non-urbanized areas; and an improved PS InSAR method was presented to estimate deformation without requiring phase unwrapping [27]. Recently, the Multi-temporal Interferometric Synthetic Aperture Radar (multi-temporal InSAR) technique [28] has been proposed and applied in monitoring surface deformation [29]. With the combination of PS InSAR and SBAS InSAR, the multi-temporal InSAR technique can simultaneously retrieve the deformation information of PSs and DSs and improve deformation information in investigated areas.

Researchers have investigated the surface subsidence in Beijing via different approaches and data. Chen et al. [6] comprehensively analyzed the subsidence situation by combining groundwater contours from 2003 to 2006 and subsidence deformation map derived from the PS InSAR technique during the same time period. Ng et al. [30] retrieved the deformation map in Beijing from 1 February 2007 to 1 November 2008, using ENVISAT ASAR and ALOS PALSAR data based on the PS InSAR technique; the results showed that the subsidence rates ranged from −115 mm/year to 6 mm/year in most areas. Hu et al. [31] investigated the spatial-temporal distribution characteristics of surface subsidence in Beijing from 2003 to 2010 via SBAS InSAR. The results showed that the maximum subsidence rates in the Tongzhou District exceeded 110 mm/year. Chen et al. [32] employed the SBAS InSAR technique to process ENVISAT ASAR images acquired from 2003 to 2010 and TerraSAR-X stripmap images collected from 2010 to 2011 to investigate surface subsidence in the Beijing region; the results indicated that the Beijing region experienced obvious surface subsidence from 2003 to 2011, with a maximum accumulative displacement of 790 mm where the maximum subsidence is located in the eastern part of Beijing (the rate exceeded 100 mm/year).

However, few previous studies have investigated the relationship between surface subsidence and groundwater changes in Beijing using both the SAR and GRACE data simultaneously. Although the spatial resolution of GRACE is approximately 300 km, it can retrieve the true amplitudes of large water storage changes in a concentrated area that is much smaller than the resolution of GRACE global harmonic solutions. Wang et al. [33] retrieved the water storage changes in the Three Gorges Reservoir (TGR) of China by GRACE solutions from April 2002 to May 2010 that agree well with the in situ measurements of TGR impounded water volume variations. Zhou et al. [34] investigated the terrestrial water storage (TWS) variations in the Poyanghu Basin using GRACE gravity data from January 2003 to March 2014, and the GRACE-based TWS variations showed a general consistency with the TWS variations from satellite altimetry. Similar studies have also used GRACE to detect mass variations within the GRACE footprint (approximately 200,000 km^2^), such as in southern Mali, Africa [35], the California Central Valley [36,37] and the North China Plain [38]. In these regions, mass variations with large variability are highly concentrated. Therefore, the signal-to-noise ratio of mass changes is high enough to be detected by GRACE. In addition, GRACE provides an alternative approach to study the contribution of groundwater changes on surface subsidence. Liu et al. [39] used GRACE and GPS data to investigate vertical deformation due to significant groundwater depletion in the North China Plain. They found the GRACE- and GPS-derived secular trends and seasonal signals coincide well.

In this paper, to increase the spatial resolution of deformation information, we first used the multi-temporal InSAR technique to retrieve the surface subsidence velocities and time series. We then adopted leveling data to verify the multi-temporal InSAR-derived results. Furthermore, we retrieved the corresponding period groundwater changes in the Beijing area using monthly GRACE data. In addition, we comprehensively analyzed the spatial-temporal characteristics of surface subsidence via groundwater changes and multi-temporal InSAR-derived subsidence information. Finally, the cross-correlation between surface subsidence and the groundwater changes were analyzed in detail.

This paper is organized as follows: Section 2 provides the information about the study area and data. Section 3 describes the details of the multi-temporal InSAR technique and the calculation model of TWS changes and groundwater storage changes from GRACE. Section 4 presents the multi-temporal InSAR-derived subsidence results. In Section 5, we validate the results by leveling data and discuss the relationship between the subsidence results and groundwater changes. The conclusions are provided in Section 6.

## 2. Study Area and Data

### 2.1. Study Area

**Figure 1:**
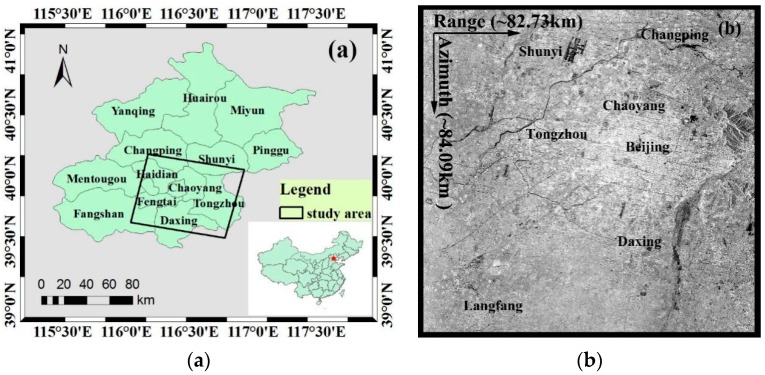
The study area in Beijing, China. The black box shown in (**a**) is the study area. (**b**) The SAR amplitude image of ENVISAT ASAR covering the study area.

Beijing (115°42′ E–117°24′ E; 39°24′ N–41°36′ N), the capital of China, is located in the North China Plain with a total area of approximately 16,410.54 km^2^ (Figure 1a). The terrain is relatively flat, and the average elevation is 43.5 m. The permanent resident population of Beijing was 21.689 million in June 2015. The plain area in Beijing is mainly the piedmont alluvial-proluvial plain formed by the joint action of the Yongding River, Chaobai River, Wenyu River, Ju River and other rivers. Multiple subsidence areas (e.g., Chaoyang and Tongzhou subsidence areas) are situated in the plain area. The range of the black box illustrated in Figure 1a is the study area, covering most of the Beijing Plain; the central latitude and longitude are 39°50′ N and 116°31′ E, respectively. Figure 1b shows that the amplitude image of ENVISAT ASAR covers the study area with a total area of approximately 6956.77 km^2^ (approximately 82.73 km in the range and 84.09 km in the azimuth).

Since the 1960s, population growth, urban construction, and industrial and agricultural development in Beijing have caused increasing exploitation of the groundwater, resulting in a gradually expanding surface subsidence range. The exploitation of groundwater in Beijing was 5.2 × 10^8^ m^3^ in 1961, and the average exploitation reached 10.79 × 10^8^ m^3^/year between 1961 and 1970. The exploitation of the groundwater was 25.59 × 10^8^ m^3^ in 1978, and the average exploitation of the groundwater reached 26 × 10^8^–28 × 10^8^ m^3^/year between 1990 and 1999 [40]. During 1999 to 2009, the groundwater exploitation remained larger than the groundwater recharge in Beijing. The annual average over-exploitation reached 9.55 × 10^8^ m^3^/year between 1999 and 2007 [41], causing a continuous and rapid decline in the groundwater level and accelerating the surface subsidence in Beijing.

### 2.2. Data

In this paper, we selected eighteen level 0 descending ENVISAT ASAR images acquired from 1 August 2007 to 29 September 2010, over Beijing to estimate the line-of-sight (LOS) average surface subsidence velocity and subsidence time series. The incidence angle of the ASAR image over the study area ranges from 17.9° in the near range to 22.8° in the far range. All of the ASAR images are in VV polarization mode with a resolution of 7.804 m in slant range and 4.750 m in azimuth. The three arc-second Shuttle Radar Topography Mission (SRTM) DEM provided by the National Aeronautics and Space Administration (NASA) was used to remove the topographic phases. Meanwhile, the DORIS orbit data released by the European Space Agency (ESA) was adopted to improve the orbit data precision of the ASAR images. To validate the results, twenty-six leveling points provided by the Beijing Institute of Surveying and Mapping were used to validate the multi-temporal InSAR-derived subsidence results, the leveling data were acquired from 2007 to 2010.

As mentioned in Section 2.1, large groundwater loss has occurred in Beijing, and the groundwater changes are mainly concentrated on the plain area of Beijing. Although the study area is much smaller than the spatial resolution of GRACE global harmonic solutions, the true amplitudes of the large groundwater changes in the study area can be retrieved by GRACE solutions with the help of hydrologic modeling. In this study, we adopted the monthly gravity fields from the Release (RL) 05 solution provided by the Center for Space Research (CSR) at the University of Texas at Austin from August 2007 to September 2010 to estimate the regional TWS changes by averaging the values in grids over the Beijing area. TWS changes at 6 grid points derived from GRACE in the region from 115°–118° E and 39°–41° N with an area of ~56,000 km^2^ were selected. Soil moisture (SM) changes from the NOAH version of the Global Land Data Assimilation System (GLDAS) [42] at a spatial resolution of 1° × 1° were used to obtain regional groundwater storage variations by subtracting them from the GRACE-based TWS changes.

## 3. Methodology

### 3.1. Multi-Temporal InSAR Technique

**Figure 2:**
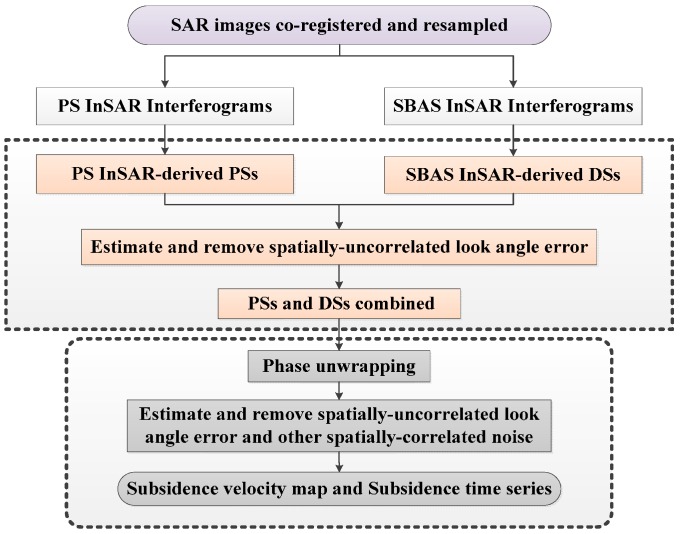
Flow diagram of the multi-temporal InSAR technique.

**Figure 3:**
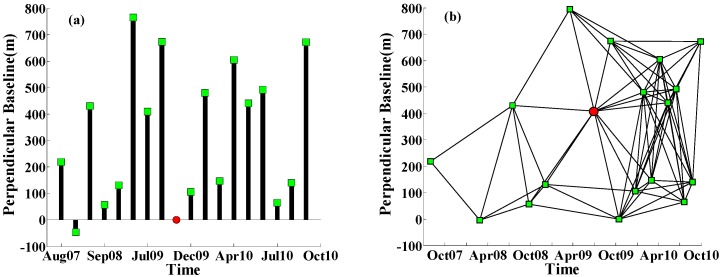
(**a**) Pairs (interferograms) of ENVISAT images processed by PS InSAR; (**b**) Pairs of ENVISAT images processed by SBAS InSAR. The red dot denotes the super master image. Both black lines in (**b**) and the black bars in (**a**) represent the pairs from which the interferograms were generated. The green square denotes the slave image.

The multi-temporal InSAR technique combines the approach of selecting high-coherence points in the PS InSAR technique and the superiorities of SBAS InSAR that retrieve high-accuracy deformation information based on multiple-master interferograms and perform better in detecting distributed scatterers. This approach first uses the PS InSAR and SBAS InSAR techniques to select the high-coherence points from images and then retrieves the deformation information via joint processing of the high-coherence point data sets. Therefore the spatial sampling of deformation information is improved. In this study, the subsidence velocity map and time series in the Beijing area were obtained by processing the 18 ENVISAT ASAR images with the multi-temporal InSAR technique. The main steps are as follows (Figure 2):

(1) The image acquired on 14 October 2009, was selected as the super master image for the interferometric combinations, and all the slave images were co-registered and resampled to the super master image.
(2) Seventeen interferograms were generated for PS analysis (Figure 3a); meanwhile, 71 pairs of small baseline differential interferograms were processed using SBAS InSAR with a perpendicular baseline shorter than 800 m and a temporal baseline less than 400 days (Figure 3b). Supposing interferogram *j* is generated from a master image and a slave image acquired at times *t_B_* and *t_A_* (*t_B_* > *t_A_*), after removing the flat earth effect and topographic phase, the interferometric phase of a pixel located at coordinates (*x,r*) in interferogram *j* can be expressed as follows [22,43]:
  (1) $$\begin{array}{l} \delta \phi_{j}(x,r)=\phi (t_{B},x,r)-\phi (t_{A},x,r)\\ \approx \phi_{def,j}(x,r)+\phi_{atm,j}(x,r)+\phi_{orb,j}(x,r)+\phi_{\theta ,j}(x,r)+\phi_{noise,j}(x,r) \end{array}$$
  where *φ*(*t_B_*,*x,r*) and *φ*(*t_A_*,*x,r*) are the phase values of the SAR images at times *t_B_* and *t_A_*, respectively. *φ_def,j_*(*x,r*) represents the deformation phase between times *t_A_* and *t_B_* in the LOS direction. *φ_atm,j_*(*x,r*) refers to the atmospheric phase error. *φ_orb,j_*(*x,r*) is the residual phase due to orbit inaccuracies. *φ_θ,j_*(*x,r*) is the residual phase due to look angle error, and *φ_noise,j_*(*x,r*) is the random noise phase.
(3) PSs were identified from the interferograms generated by PS InSAR using the algorithm proposed by Hooper et al. [43]. We retrieved DSs from small baseline interferograms in the same way that PS pixels were retrieved, i.e., following the algorithm of Hooper et al. [43]. The spatially-uncorrelated look angle error term, which includes contributions from both spatially-uncorrelated height errors and deviations of the pixel’s phase center from its physical center, was estimated and removed after selecting the PSs and DSs. After calculating the equivalent small baseline interferometric phase for the PSs by recombining the single-master interferometric phase, the small baseline interferometric phases from both selected PSs and DSs were combined. If a pixel occurred in both data sets, a weighted mean value for the phase was calculated by summing the complex signal from both data sets [28].
(4) The phase was unwrapped by a minimum-cost flow algorithm. Then, the spatially-correlated look angle error and other spatially-correlated noise were estimated and subtracted from the differential phase. Next, the deformation rates of the study area were obtained by a least-squares algorithm because no isolated interferogram clusters in the analysis. After obtaining the deformation rates, according to the time span between ASAR images, the corresponding deformation time series was derived.

### 3.2. Calculation of TWS and Groundwater Storage Changes from GRACE

Based on the method of Wahr et al. [44], TWS changes can be recovered from the GRACE temporal gravity field, and the calculation model of TWS changes can be expressed by the equivalent water height (EWH) as follows [44,45,46]:

(2) $$EWH\left(\theta ,\lambda \right)=\frac{a\rho_{ave}}{3\rho_{water}}\sum_{l=0}^{\infty }\sum_{m=0}^{l}{\bar{P}}_{lm}\left(\cos\theta \right)\frac{2l+1}{1+k_{l}}\left(\Delta C_{lm}\cos\left(m\lambda \right)+\Delta S_{lm}\sin\left(m\lambda \right)\right)$$

where *a* (63,781,363.3 m) and *ρ_ave_* (5517 kg/m^3^) are the average radius and density of the Earth, respectively; *ρ_water_* is the density of water (1000 kg/m^3^); ${\bar{P}}_{lm}$is the fully normalized associated Legendre function; *k_l_* represents the degree-l Love numbers; Δ*C_lm_* and Δ*S_lm_* are the variations in the spherical harmonic coefficients of the gravity field with respect to the average gravity field for the period ranging from August 2007 to September 2010; and *θ* and *λ* are the co-latitude and longitude, respectively.

Monthly gravity fields from RL05 (at degree-l and order-m 60) provided by the CSR for the period of August 2007 to September 2010 were used to calculate the TWS changes. The data processing, which followed the methods of Luo et al. [47], included the following steps: (1) In GRACE observed harmonics coefficients, the degree-2 zonal harmonics were not well estimated because the GRACE orbit geometry is less sensitive to the coefficient of the gravity field. Therefore, degree-2 zonal C20 time series were replaced by analyzed Satellite Laser Ranging (SLR) data [48]; (2) Due to the correlated errors among certain spherical harmonics coefficients and other errors found in GRACE spherical harmonic coefficients, a hybrid filtering scheme that combined de-correlation filter P3M6 (at spherical harmonics orders 6 and above, a degree 3 polynomial is fitted via least squares and is removed from even and odd coefficients pairs) [49] and a 300 km Fan filter [50] were applied to reduce noise in the GRACE data. The computed model can be expressed as follows [51,52]:

(3) $$\begin{array}{l} EWH\left(\theta ,\lambda \right)=\frac{a\rho_{ave}}{3\rho_{water}}\sum_{l=0}^{\infty }\frac{2l+1}{1+k_{l}}W_{l}\sum_{m=0}^{l}{\bar{P}}_{lm}\left(\cos\theta \right)\cdot W_{m}\\ \left(\Delta {\tilde{C}}_{lm}\cos\left(m\lambda \right)+\Delta {\tilde{S}}_{lm}\sin\left(m\lambda \right)\right) \end{array}$$

where *W_l_* and *W_m_* are the smoothing kernel functions associated with order and degree, respectively. That is: *W*_0_ = 0, $W_{1}=\frac{1+e^{-2b}}{1-e^{-2b}}-\frac{1}{b},\;$ $W_{N+1}=-\frac{2n+1}{b}W_{n}+W_{n-1},\;$, and $b=\frac{\ln2}{1+\cos\left(\frac{r}{a}\right)};r$ is the filtering radius; and $\Delta {\tilde{C}}_{lm}$ and $\Delta {\tilde{S}}_{lm}$ are the variations in the spherical harmonic coefficients of the gravity field in Equation (2) after decorrelation filtering.

The GLDAS-based SM was initially processed in the same way as the GRACE data (i.e., model grids were transferred to spherical harmonic coefficients, truncated at degree and order 60, and a de-correlation filter and a 300 km Fan filter were applied) for consistency with the GRACE resolution. Then, groundwater storage changes were computed by subtracting SM changes from GRACE TWS changes [53].

## 4. Results

**Figure 4:**
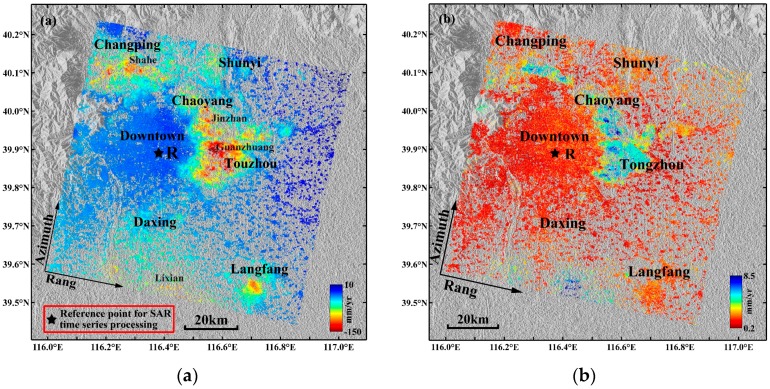
(**a**) The LOS average subsidence velocity map derived by the multi-temporal InSAR technique superimposed on the shaded relief topography of the Beijing area; the black star denotes the reference point. (**b**) The standard deviations of average subsidence velocities.

The LOS average subsidence velocity map in the study area derived by the multi-temporal InSAR technique is shown in Figure 4a, where the negative values indicate that the surface is moving away from the satellite (i.e., subsidence in the LOS direction) and the positive values indicate surface uplift. The standard deviations of the average subsidence velocities in the study area are shown in Figure 4b. The standard deviations were obtained by computing the deviations of the linear fitting of the velocities. If a PS/DS point showed a strong non-linear motion, it resulted in a large residual with respect to the linear model, i.e., in a high standard deviation. Based on the collected leveling data, a stable point called R (Figure 4a) was selected as a reference point in the study area; all of the monitoring points’ average subsidence velocities in the deformation velocity field are related to the reference point.

As shown in Figure 4a, in the study area, uneven subsidence is prominent. The five significant subsidence areas are located in Changping, Shunyi, Chaoyang, Tongzhou and Daxing Districts. Additionally, a pronounced subsidence area located in Langfang City can be identified, which is adjacent to Beijing and has a maximum velocity exceeding 75 mm/year. The main subsidence areas in Beijing are distributed in the Chaobai River, Wenyu River and Yongding River alluvial-proluvial fan plains. The Changping, Shunyi, Chaoyang and Tongzhou subsidence areas are mainly distributed along the Wenyu River, Chaobai River, Qing River, and Tonghui River basins, and these subsidence areas gradually connect into a continuous area and constitute the “North” subsidence area. The “North” subsidence area gradually expands toward the northern part of Shunyi, the eastern part of Chaoyang, and the eastern and southern parts of Tongzhou.

In addition, a new subsidence area has formed east of the study area, i.e., the Yanjiao Town subsidence area, and some of the subsidence velocities in this area have reached 50 mm/year. The Daxing subsidence area is mainly distributed in the Yongding River coast and constitutes the “South” subsidence area. The subsidence range of the “South” subsidence area is expanding markedly in a northward direction.

The surface subsidence in downtown Beijing (e.g., Haidian, Fengtai and Shijingshan Districts and the central urban area) is small and relatively stable, and most of the subsidence velocities range from −10 mm/year to 10 mm/year. Compared with the Changping, Chaoyang and Tongzhou subsidence areas, subsidence velocities in the Shunyi and Daxing subsidence areas are lower. The most serious subsidence area in Daxing District is Lixian Town, where the maximum subsidence velocity exceeds 70 mm/year. The maximum subsidence velocity in the Shunyi subsidence area reaches 60 mm/year. Multiple subsidence funnels have formed in Changping District, among which the Shahe and Shangzhuang Town subsidence centers are more serious, with an annual subsidence velocity exceeding 80 mm/year. The subsidence in Laiguangyin, Sunhe and Jinzhan Townships in Chaoyang District are substantial; the maximum subsidence velocity in the Sunhe subsidence center exceeds 80 mm/year, and the most serious subsidence area is the Jinzhan subsidence area in which the maximum subsidence velocity exceeds 130 mm/year. Guanzhuan, Sanjianfang and Dougezhuang Townships are the most severe subsidence areas in Tongzhou District. Subway Batong Line, Subway Line 6 and Huitong River run through these areas along an east-west direction, and these areas include several highways and railways. In addition, the groundwater exploitation in these areas is serious. Therefore, it is expected that the surface subsidence in Tongzhou District is not only affected by the surface dynamic load and the utilization of underground space but also by the groundwater over-exploitation and geological structure. Multiple subsidence centers are located in the area, which includes the metro, highways, railway and rivers, and the maximum annual average subsidence velocities range between 140 mm/year and 150 mm/year.

**Figure 5:**
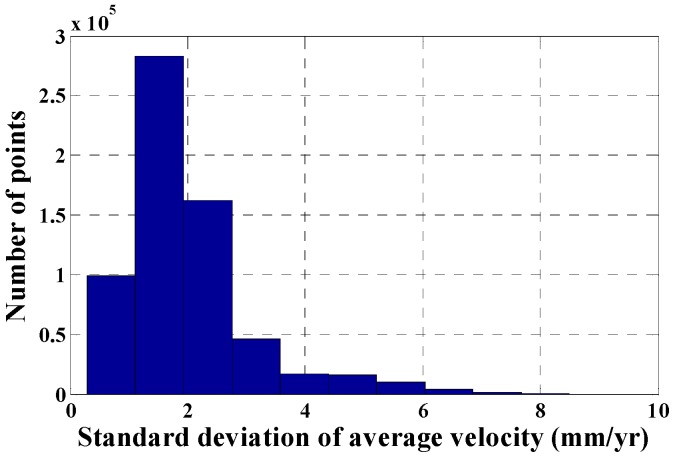
Distributions of the standard deviations of the average velocities (from Figure 4b).

Figure 4b shows that the standard deviations of the average velocities from all monitoring points derived by the multi-temporal InSAR technique range from 0.2 mm/year to 8.5 mm/year; the monitoring points with larger standard deviations are mainly distributed in the serious subsidence areas (e.g., Tongzhou, Chaoyang and Changping Districts); the standard deviations of the average velocities of most monitoring points distributed in the downtown area of Beijing are less than 2 mm/year. The statistical analysis of the standard deviations of all monitoring points (Figure 5) suggests that the standard deviations of the average velocities of 96.81% monitoring points are less than 5 mm/year and 88.46% are less than 3 mm/year; the standard deviation of the subsidence results derived by the multi-temporal InSAR technique is 1.99 mm/year. Therefore, the surface subsidence monitoring accuracy using the multi-temporal InSAR technique is high.

## 5. Discussion

### 5.1. Validation with Leveling

**Figure 6:**
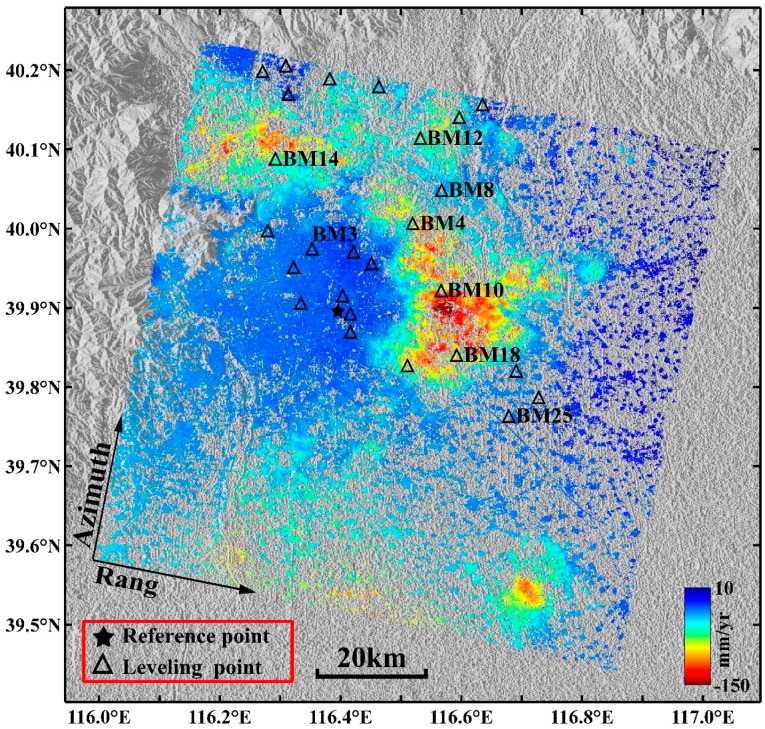
Location of leveling points plotted on the average subsidence velocity map of the study area. The black star denotes the reference point, and a triangle represents a leveling point.

To verify the reliability of the multi-temporal InSAR-derived results, we compared and analyzed the multi-temporal InSAR-derived results with the leveling data provided by the Beijing Institute of Surveying and Mapping; the location distribution of all leveling points are illustrated in Figure 6. Several steps were performed for the validation: (1) the overlapping period data of the leveling and ENVISAT ASAR image data were selected; (2) the vertical deformation of all leveling points were projected into the LOS direction; and (3) the initial subsidence on 1 August 2007, was assumed to be zero. Because there are few opportunities for a leveling point and the corresponding PS point to be located in the same place, two different methods were adopted to extract the subsidence of the PS point corresponding to the nearest leveling point. In the first method (Kriging method), the spherical variogram model (detailed information shown in Appendix A) was computed based on the multi-temporal InSAR-derived PS point subsidence. Based on the spherical variogram model, Kriging was employed to estimate the subsidence of the corresponding PS point located in the same location with respect to the leveling point. For the second method (average method), the average subsidence rates of the PS points that lie within 200 m of the leveling point were used as the subsidence of the corresponding PS point.

**Figure 7:**
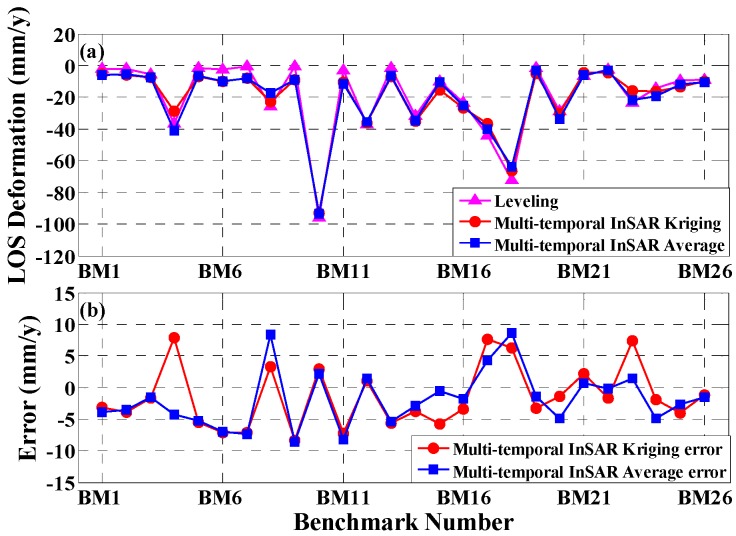
(**a**) Comparison between multi-temporal InSAR- and Leveling-derived results. The blue squares represent the results by the Kriging method, the red circles represent the results using the average method and the purple triangles represent the results from leveling. (**b**) Differences between multi-temporal InSAR- and Leveling-derived results.

We adopted the Kriging and average methods to extract the average subsidence velocity of a PS point located at the same location as a leveling point; the results are shown in Figure 7. Figure 7a shows that the deformation changes of the three curves are approximately the same, indicating that the average subsidence velocities extracted by the two methods agree well with the results obtained from the leveling data. Moreover, the differences between multi-temporal InSAR and leveling are within ±10 mm/year, as shown in Figure 7b. We quantitatively compared the average subsidence velocities of the 26 points (i.e., BM1–BM26) derived using the multi-temporal InSAR technique and leveling (results listed in Table 1). For the Kriging method, the mean error is 1.44 mm/year, the root-mean-square error (RMSE) is 4.97 mm/year, the maximum difference is 8.33 mm/year and the minimum difference is 1.01 mm/year, while the mean error, RMSE, MAX and MIN achieved from the average method are 1.90 mm/year, 4.78 mm/year, 8.60 mm/year, and 0.18 mm/year, respectively. The accuracies of the two methods are similar. Therefore, the validation results indicate that the average subsidence velocities obtained by the multi-temporal InSAR technique are highly consistent with those derived by leveling, and the multi-temporal InSAR technique can satisfy the subsidence monitoring requirements in the study area.

**Figure 8:**
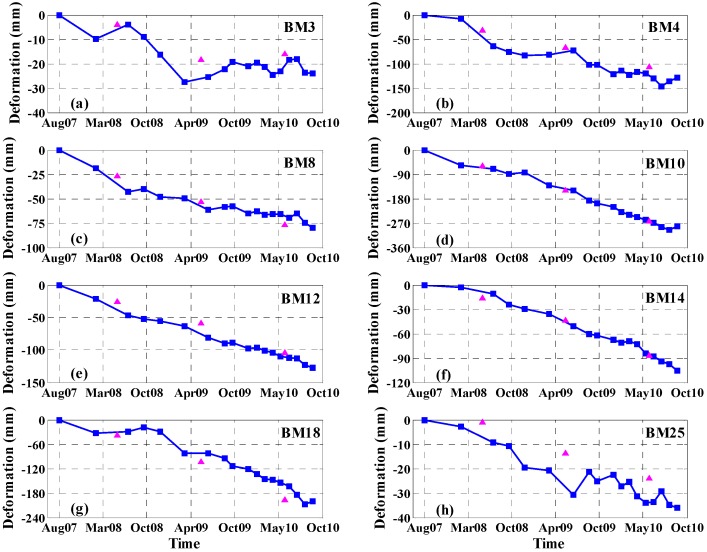
Comparison of eight benchmarks marked in Figure 6 between the subsidence time series of multi-temporal InSAR (blue squares) and leveling (purple triangles) data.

The leveling data were acquired only once during the summers of 2007, 2008, 2009 and 2010. To analyze the temporal evolution of surface subsidence in the study area, we selected eight leveling points (i.e., BM3, BM4, BM8, BM10, BM12, BM14, BM18 and BM25 illustrated in Figure 6) and the corresponding PS points to compare and analyze their subsidence time series. The results are shown in Figure 8.

As shown in Figure 8, the multi-temporal InSAR-derived results show nonlinear subsidence. The selected points show different amounts of subsidence depending on their location. Leveling point BM3 and the corresponding PS point are located in the downtown area of Beijing (Figure 6), where the accumulated subsidence values of the two different measuring methods are less than 30 mm (Figure 8a). Meanwhile, the subsidence is nearly steady after April 2009, which is related to the stringent restrictions on groundwater exploitation in the urban area and the small compressibility of most surface sediments (e.g., coarse particle sand, gravel, and cobble) in the western part of Beijing. Among the selected points, BM10 and BM18 are located in the most serious subsidence area, the Tongzhou District (Figure 6). The subsidence of these pairs continues to increase, and the accumulated subsidence has exceeded 200 mm at both locations. At BM10 and BM18, the results derived by multi-temporal InSAR and leveling coincide well, as shown in Figure 8d,g. For other points, the multi-temporal InSAR-derived results are also consistent with the leveling-derived results. The comparative analysis indicates that the subsidence values acquired by leveling agree well with the multi-temporal InSAR-derived results.

### 5.2. Comparison between Subsidence Changes and Groundwater Changes

**Figure 9:**
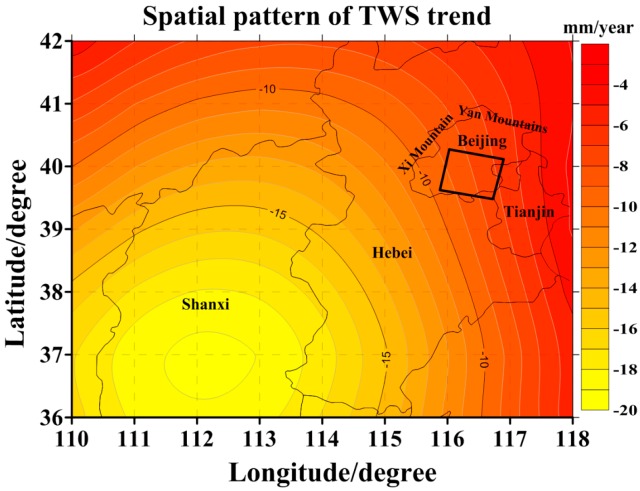
Spatial pattern of the TWS trend across Beijing, Tianjin, Hebei and Shanxi as estimated from GRACE data from August 2007 to September 2010. The black square is the location and size of the study area.

**Figure 10:**
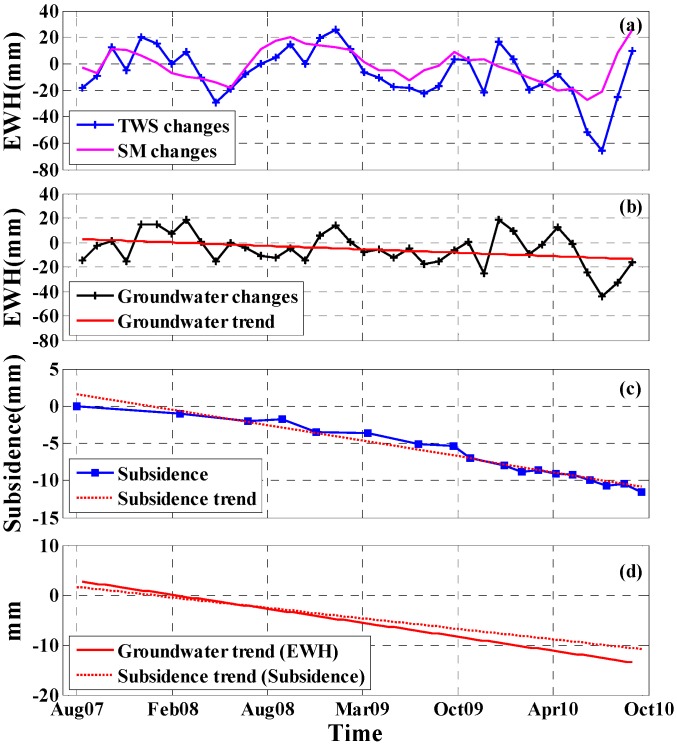
(**a**) TWS changes (blue curve) estimated from the GRACE data and SM changes (purple curve) estimated by GLDAS_NAOH in the study area; (**b**) Groundwater changes (black curve) and groundwater trend (red line); (**c**) Average subsidence changes (blue curve) estimated using the multi-temporal InSAR technique and the average subsidence trend (red dotted line) in the study area; (**d**) Comparison between the groundwater trend (red line) and average subsidence trend (red dotted line).

To analyze the correlation between surface subsidence and groundwater changes in Beijing, the monthly gravity fields from RL05 (at degree-l and order-m 60) between August 2007 and September 2010 were first used to estimate the TWS changes in the study area. The long-term trend in TWS changes estimated from GRACE is shown in Figure 9. The mean long-term trend of TWS changes in the study area is −8.0 mm/year (in terms of EWH). The time series of the regional TWS changes in the study area were achieved by averaging the values in the study area. This result is illustrated in Figure 10a (blue curve) and shows pronounced seasonal variations.

The TWS changes estimated from the GRACE data include primarily groundwater changes, surface water changes, soil moisture (SM) changes, and ice and snow water changes. Beijing is located in a warm temperate and semi-humid continental monsoon climate zone. Thus, the snow water component is typically small, and its contribution to the long-term TWS trend in the Beijing area is negligible. Nearly all of the major rivers in Beijing are dammed for municipal and industrial use; no reservoirs are located in the study area. The surface water reservoir storage changes in the study area have little influence on long-term TWS changes during the study period [54]. Because our aim is to derive the long-term trend in groundwater changes, the impact of the surface water component on TWS changes is beyond our consideration.

The Yan Mountains and Xi Mountain are located in the northern and western parts of Beijing (Figure 9), respectively, and the area of these mountains accounts for approximately 60 percent of the area of Beijing. In Figure 4a, it was shown that the subsidence velocities are significantly larger than the surface uplift. Additionally, because the mountains have limited capacity to store groundwater and groundwater exploitation in Beijing is mainly concentrated in the plain area, the TWS change estimated from the GRACE data in the selected area results largely from groundwater depletion in the plain region of Beijing. Although the area for the multi-temporal InSAR data is much smaller than the footprint of GRACE data, the prior conclusion means that it is reasonable to compare the GRACE-based groundwater trend with the multi-temporal InSAR-derived surface subsidence trend.

The SM changes from August 2007 to September 2010 in the Beijing area are shown in Figure 10a (purple curve). After removing the SM changes, the TWS changes in the Beijing area were found to be dominated by groundwater, which agrees with the intensive groundwater consumption reported in previous studies [55,56]. Therefore, groundwater changes were estimated as the difference between the GRACE-based TWS changes and the GLDAS-based SM changes, which decreased gradually during the study period (black curve in Figure 10b). The red line in Figure 10b, which was derived by the linear fitting method, reflects the long-term trend in groundwater changes in the study area. We took an average of all multi-temporal InSAR pixels to obtain the average subsidence time series for the study area, with the result (blue curve) shown in Figure 10c. The average subsidence presents a nonlinear declining tendency with seasonal variability. We adopted the linear fitting method to deduct the seasonal variability in the average subsidence and obtained the relatively long-term trend of surface subsidence in the study area, as shown in Figure 10c (red dotted line).

Figure 10d shows that both the groundwater changes and subsidence changes in the study area exhibit a continuously decreasing tendency from August 2007 to September 2010. In addition, the GRACE-based groundwater decreases at a rate of 5.3 mm/year (in terms of EWH), while the multi-temporal InSAR-derived surface subsidence trend is 3.9 mm/year (in terms of subsidence). The long-term decreasing trend in both the groundwater changes and average subsidence show a relatively high consistency. A comparative analysis shows that the groundwater decline and surface subsidence in the Beijing area are consistent. The groundwater over-exploitation seriously affects the stability of the surface in Beijing.

### 5.3. The Correlation between Surface Subsidence and Groundwater Depression

**Figure 11:**
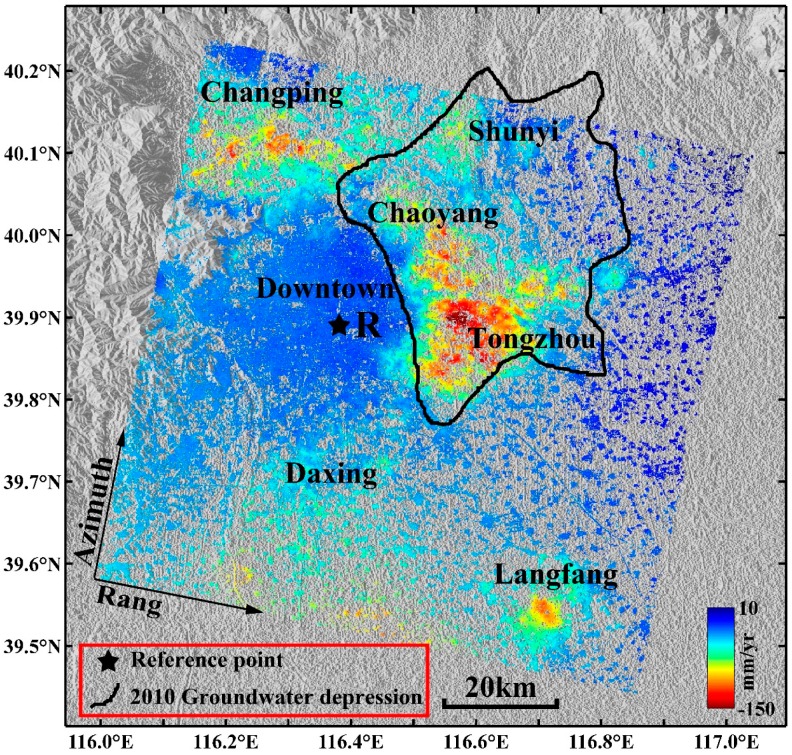
Distribution of the main groundwater depression (black curve) [58] and the LOS average subsidence velocity in the study area.

Groundwater is an important factor for maintaining soil stress balance. Groundwater over-exploitation may destroy the original soil stress balance and lead to surface subsidence. To further analyze the correlation between the surface subsidence and groundwater exploitation in the Beijing area, we compared and analyzed the spatial distribution of the average subsidence velocity from August 2007 to September 2010 and the groundwater depression cone of the main mining layer in 2010 in the Beijing area; the result is shown in Figure 11. The groundwater depression is mainly distributed in the eastern and northeastern parts of downtown Beijing, and the more serious subsidence areas (i.e., the Shunyi, Chaoyang and Tongzhou subsidence areas) are primarily located in the groundwater depression cone. The subsidence funnel and the groundwater depression are consistent to some extent, although the distribution range of the subsidence funnel is larger than that of the groundwater depression. The surface subsidence in Beijing is mainly from the deformation of the compressible deposits. Under the same stress condition, areas with higher compressible deposits are more prone to surface subsidence. The compressible sediments in the western part of Beijing (e.g., downtown) are mainly coarse-particle sand, gravel, and cobble with small compressibility, while the aquifers in the eastern, northern and southern regions (e.g., Changping, Chaoyang, Tongzhou and Daxing) are mainly composed of silt clays and fine sand characterized by thick compressible sediments [57]. The uneven surface subsidence is strongly influenced by the thickness variability of the compressible sediments, and the serious surface subsidence is generally distributed over thick compressible sediments. Thus, although the formation and development of surface subsidence in the Beijing area are mainly caused by groundwater over-exploitation, the formation and development of surface subsidence are also affected by the thickness of the compressible deposits, the hydrogeological condition, stratum lithology, and shallow surface space utilization.

## 6. Conclusions

In this study, we adopted the multi-temporal InSAR technique to derive subsidence information for Beijing from 1 August 2007 to 29 September 2010. The surface subsidence and the relationship between the surface subsidence and groundwater changes in Beijing were analyzed by combining ENVISAT ASAR data, leveling data, GRACE data, etc. The main conclusions are as follows:

(1) The surface subsidence in Beijing is notably uneven. Five significant subsidence areas that exist in Beijing are Changping, Shunyi, Chaoyang, Tongzhou and Daxing, among which Tongzhou is the most serious subsidence area, with a maximum velocity exceeding 140 mm/year. The subsidence in the downtown area of Beijing is relatively stable, where most subsidence velocities are less than 10 mm/year. Furthermore, the surface subsidence presents nonlinear deformation.
(2) A statistical analysis of the standard deviations of the average velocities indicated that 96.81% of the monitoring point standard deviations are less than 5 mm/year, and the standard deviation of the multi-temporal InSAR-derived subsidence in the study area is 1.99 mm/year. In addition, the multi-temporal InSAR-derived results were validated with leveling data. The mean error and RMSE are 1.44 mm/year and 4.97 mm/year, respectively, demonstrating that the multi-temporal InSAR technique is effective for surface subsidence monitoring in Beijing.
(3) The groundwater changes derived from the GRACE data in Beijing show a decreasing tendency and profound obvious seasonal variability. Based on the average subsidence of the study area, the long-term decreasing trends in groundwater and average subsidence are consistent. In addition, the spatial distribution of the subsidence funnel only partially overlaps the groundwater depression region. The formation and development of the surface subsidence in Beijing are seriously affected by groundwater over-exploitation.

## Figures and Tables

**Table 1:** Comparison of the average subsidence velocities between multi-temporal InSAR- and Leveling-derived results.

Method	Mean Error (mm/year)	RMSE (mm/year)	MAX (mm/year)	MIN (mm/year)
Kriging	1.44	4.97	8.33	1.01
Average	1.90	4.78	8.60	0.18

**Figure A1:**
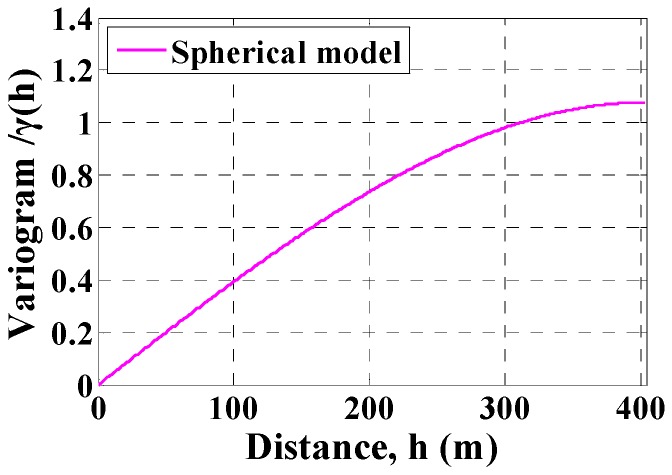
The spherical variogram model.

## Appendix A

In this study, the Kriging method was implemented through ArcGIS 10.2 software and its geostatistical analyst extension to estimate the subsidence of a PS point corresponding to the nearest leveling point. Based on the multi-temporal InSAR-derived PS point subsidence, the spherical variogram model γ(h) was obtained as follow:

(A1) $$\gamma (h)=\left\{ \begin{array}{cc} 0, & h=0\\ 1.077\times \left(\frac{3h}{2\times 402.338}-\frac{h^{3}}{2\times 402.388^{3}}\right), & 0<h\leq 402.338\\ 1.077, & h>402.338 \end{array}\right.$$

where *h* is the distance in meters. In the spherical variogram model, the nugget, sill and major range are 0, 1.077 and 402.338, respectively. Figure A1 shows the spherical variogram model.

## Appendix B

The acronyms used in the text are listed in Table B1.

**Table B1:** List of acronyms used in the text.

Acronym	Full Name
CSR	Center for Space Research
DSs	Distributed scatterers
DInSAR	Differential Interferometric Synthetic Aperture Radar
ESA	European Space Agency
EWH	Equivalent water height
GLDAS	Global Land Data Assimilation System
GRACE	Gravity Recovery and Climate Experiment
GNSS	Global Navigation Satellite System
InSAR	Interferometric Synthetic Aperture Radar
ISBAS	Intermittent Small Baseline Subset
LOS	Line of Sight
Multi-temporal InSAR	Multi-temporal Interferometric Synthetic Aperture Radar
NASA	National Aeronautics and Space Administration
PS InSAR	Persistent Scatterer Interferometric Synthetic Aperture Radar
PSs	Point-like stable reflectors
QPS	Quasi Persistent Scatterer
RMSE	Root Mean Square Error
RL05	Release 05
SRTM	Shuttle Radar Topography Mission
SLR	Satellite Laser Ranging
SM	Soil Moisture
SBAS InSAR	Small Baseline Subset Interferometric Synthetic Aperture Radar
TGR	Three Gorges Reservoir
TWS	Terrestrial Water Storage
